# Analysis of right anterolateral impacts: the effect of trunk flexion on the cervical muscle whiplash response

**DOI:** 10.1186/1743-0003-3-10

**Published:** 2006-05-16

**Authors:** Shrawan Kumar, Robert Ferrari, Yogesh Narayan, Edgar Vieira

**Affiliations:** 1 Department of Physical Therapy, Faculty of Rehabilitation Medicine, University of Alberta, Edmonton, Alberta, T6G 2G4, Canada; 2 Department of Medicine, University of Alberta, Edmonton, Alberta, T6G 2B7, Canada

## Abstract

**Background:**

The cervical muscles are considered a potential site of whiplash injury, and there is a need to understand the cervical muscle response under non-conventional whiplash impact scenarios, including variable body position and impact direction. There is no data, however, on the effect of occupant position on the muscle response to frontal impacts. Therefore, the objective of the study was to measure cervical muscle response to graded right anterolateral impacts.

**Methods:**

Twenty volunteers were subjected to right anterolateral impacts of 4.3, 7.8, 10.6, and 12.8 m/s^2 ^acceleration with their trunk flexed forward 45 degrees and laterally flexed right or left by 45 degrees. Bilateral EMG of the sternocleidomastoids, trapezii, and splenii capitis and acceleration of the sled, torso, and head were measured.

**Results and discussion:**

With either direction of trunk flexion at impact, the trapezius EMGs increased with increasing acceleration (*p *< 0.05). Time to onset of the electromyogram and time to peak electromyogram for most muscles showed a trend towards decreasing with increasing acceleration. With trunk flexion to the left, the left trapezius generated 38% of its maximal voluntary contraction (MVC) EMG, while the right trapezius generated 28% of its MVC EMG. All other muscles generated 25% or less of this measure (25% for the left splenius capitis, 8% for the right splenius capitis, 6% for the left sternocleidomastoid, and 2% for the left sterncleidomastoid). Conversely, with the trunk flexed to the right, the right trapezius generated 44% of its MVC EMG, while the left trapezius generated 31% of this value, and all other muscles generated 20% or less of their MVC EMG (20% for the left splenius capitis, 14% for the right splenius capitis, 4% for both the left and right sternocleidomastoids).

**Conclusion:**

When the subject sits with trunk flexed out of neutral posture at the time of anterolateral impact, the cervical muscle response is dramatically reduced compared to frontal impacts with the trunk in neutral posture. In the absence of bodily impact, the flexed trunk posture appears to produce a biomechanical response that would decrease the likelihood of cervical muscle injury in low velocity impacts.

## Background

Whiplash injury is an important health problem with a significant economic and health burden [1]. There has been considerable research on the cervical response to rear-end impacts using volunteers [2-18], but much less research with volunteers in frontal impacts, most of the early frontal impact studies being done with military personnel [19-24]. We know much less, therefore, about the mechanism of whiplash injury in frontal collisions. This is despite the fact that a recent large epidemiological study has confirmed that frontal collisions are as common a cause of whiplash claims as rear-end collisions [25].

We have applied a methodology which combines surface EMG and extrapolations through regression based on very-low velocity impacts to the problem of frontal impacts. This has been done with straight-on frontal impacts [24], and recently in this journal we also reported on the effect of head rotation in anterolateral impacts specifically [26]. Using this approach, the regression models are thus far in good agreement with the available data that has been gathered in previous, small studies of higher velocity impacts [27]. It has also been shown that if the subject is expecting an impact, this mitigates the risk of injury [18].

The reality is that vehicle occupants are not always positioned in this neutral position at the time of impact. Foret-Bruno [28] has reviewed that whiplash victims may be in the trunk-flexed position, and that, at least from dummy experiments, this may increase the risk of injury in a frontal impact, not only from impact with the vehicle interior, but through effects of increased cervical extension when the occupant is seated with most of the torso away from the seat and rebounds into the seat after the impact. There is yet, however, no volunteer data which examines the cervical responses of volunteers when they are not seated in the standard, neutral head and trunk posture.

Since we have recently reported in this journal on the effect of head rotation in anterolateral impacts, it was of interest to keep the impact variables constant and determine whether trunk flexion itself in anterolateral impacts will increase or decrease the EMG activity, and how. We thus undertook a study to assess the cervical muscle response in right anterolateral impacts, but with the trunk flexed to either the left or right (to mimic circumstances of "out-of-position" vehicle occupants) at the time of impact.

## Methods

The methods for this study of frontal impacts with trunk flexion are the same as those used previously for frontal impact studies with the subject in either neutral posture and/or with head rotation [24,26,29,30]. Twenty healthy, normal subjects (10 males and 10 females) with no history of whiplash injury and no cervical spine pain during the preceding 12 months volunteered for the study. The 20 subjects had a mean age of 23.6 ± 3.0 years, a mean height of 172 ± 7.7 cm, and a mean weight of 69 ± 13.9 kg. The subjects were all right-hand dominant. The study was approved by the University Research Ethics Board.

**Figure 1:**
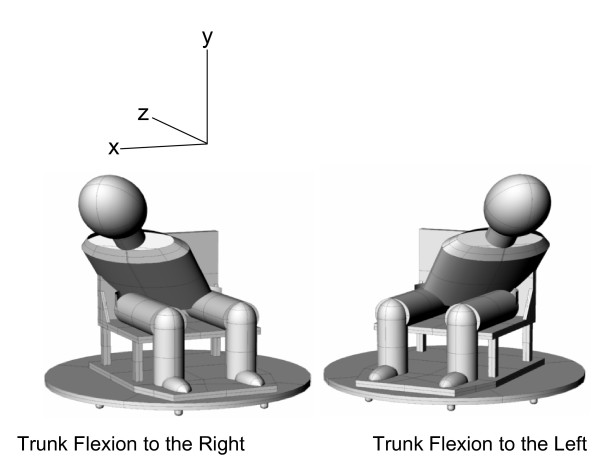
Illustration of the positioning of the subjects prior to frontal whiplash-type impacts.

The sled device is shown in this journal in the previous publication [26]. Subjects were then exposed to right anterolateral impacts with their trunk flexed forward and to either their left and right at accelerations of 4.3, 7.8, 10.6, and 12.8 m/s^2 ^generated in a random order by a pneumatic piston. The subjects were asked to assume a position of trunk flexion (forward and lateral) and to look down at their right or left foot. We positioned each of the volunteers in 45 degrees flexion and 45 degrees rotation either to the left or to the right (see Fig. 1). We did not use any blocking of visual or auditory cues, which is comparable to the "expected" impact data we had gathered previously [24,26], but the impact severity and posture positions were randomly varied between the 4 levels of acceleration. Each subject effectively underwent 4 levels of accelerative impacts under two conditions of trunk flexion, for one direction of impact (a total of 8 impacts). The acceleration was delivered in a way that mimicked the time course seen in motor vehicle collisions and occurred fast enough to produce eccentric muscle contractions. Subjects were asked to report any headache or other aches or discomfort they experienced in the days following the impacts for a period of up to 6 months. None were reported.

## Results and discussion

### Head acceleration

As anticipated, an increase in applied acceleration resulted in an increase in excursion of the head and accompanying accelerations (p < 0.05). The accelerations in these impacts were not associated with any reported symptoms in the volunteers following the experiment and up to 6 months later.

### Electromyogram amplitude

**Figure 2:**
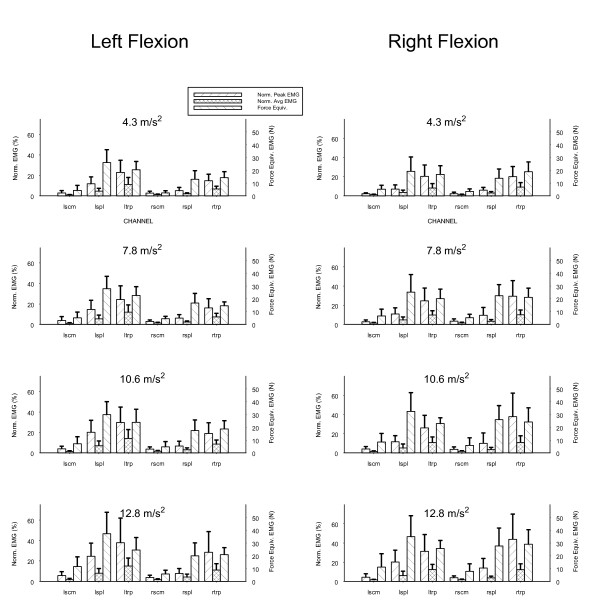
Trunk flexed to left and right. Normalized peak and average electromyogram (EMG) (percentage of isometric maximal voluntary contraction), force equivalent of EMG (N), and applied acceleration. LSCM, left sternocleidomastoid; RSCM, right sternocleidomastoid; LSPL, left splenius capitis; RSPL, right splenius capitis; LTRP, left trapezius; RTRP, right trapezius.

In a right anterolateral impact, with the trunk flexed 45 degrees to the right or left, the trapezius muscle ipsilateral to the direction of trunk flexion shows the greatest EMG response (*p *< 0.05). The normalized EMG for the sternocleidomastoid (SCM), splenius capitis (SPL) and trapezius (TRP) muscles are shown in Figure 2. At a peak acceleration of 12.8 m/s^2^, for example, with the trunk flexed to the right, the right trapezius generated 44% of its maximal voluntary contraction electromyogram, while all other muscles generated 31% or less of this variable (31% for the left trapezius, 20% for the left splenius capitis, 14% for the right splenius capitis, 4% for both the left and right sternocleidomastoids). When the trunk is flexed to the left, under these same conditions, the results are reversed even though the impact direction remains right anterolateral. When flexed to the left, the left trapezius generated 38% of its maximal voluntary contraction electromyogram, with 28% of the maximal voluntary contraction for the right trapezius, and 25% or less for the remaining muscles (25% for the left splenius capitis, 8% for the right splenius capitis, 6% for the left sternocleidomastoid, and 4% for the left sterncleidomastoid).

As the level of applied acceleration in the impact increased, the magnitude of the EMG recorded from the trapezius ipsilateral to the trunk flexion increased progressively and disproportionately compared to other muscles (*p *< 0.05). Compared to the state of the head and trunk in neutral posture, trunk flexion significantly reduces the trapezius EMG response (p < 0.05) for all conditions of flexion except for the right trapezius muscle in right trunk flexion, where the findings are equivalent to those in neutral trunk posture.

The time to onset of the sled, torso, and head acceleration showed a trend (p > 0.05) decreased with increased applied acceleration. Similarly, the time to onset of the EMG shows a trend (p > 0.05) for all muscles to decrease with increased applied acceleration. The times at which peak EMG occurred for all the experimental conditions showed a trend to earlier times of peak activity with increasing acceleration, though this again did not reach statistical significance.

The relationship between the force equivalent EMG response of each muscle and the head acceleration are shown in Table 1. To obtain the force equivalency of a muscle response due to impact, we first performed a linear regression analysis on the graded EMG data obtained in the maximal voluntary contraction trials. This resulted inan equation for force/emg ratio. EMG values from each muscle as measured in this impact study were then entered into the equation, giving us a force equivalent value (Newtons) for each muscle as shown in Table 1. The kinematic responses show that very-low velocity impacts produce less force equivalent than the maximal voluntary contraction for the same subject, and thus this experimental approach allows us to gather valuable data without exposing subjects to any foreseeable injury. The head accelerations were correspondingly lower than the sled accelerations in this experiment. For very-low velocity impacts, this is to be expected, as it is usually only when the sled acceleration exceeds 5 g's that head acceleration begins to exceed sled acceleration. This experiment involved less than 2 g accelerations.

**Table 1:** Mean Force Equivalents (Newtons, N) and Mean Head Accelerations at Time of Maximal EMG in Direction of Travel for Right Anterolateral Impact.

		Force Equivalents for Muscle (N)					
		
		Sternocleidomastoid		Splenius Capitis		Trapezius	
				
Sled Acceleration (m/s^2^)	Head Acceleration (m/s^2^)	Left	Right	Left	Right	Left	Right
Right Trunk flexion							
4.3	1.9 (0.9)	5 (3)	4 (2)	19 (11)	14 (7)	17 (7)	19 (8)
7.8	2.7 (1.4)	7 (5)	5 (3)	25 (14)	23 (9)	20 (8)	21 (7)
10.6	3.5 (0.9)	9 (7)	6 (6)	32 (15)	26 (11)	23 (5)	24 (11)
12.8	5.5 (2.7)	11 (10)	8 (6)	35 (16)	28 (14)	26 (6)	29 (11)
Left Trunk flexion							
4.3	2.2 (0.9)	4 (4)	2 (2)	26 (10)	13 (7)	20 (7)	14 (5)
7.8	3.4 (1.4)	5 (5)	5 (2)	28 (10)	17 (8)	23 (7)	15 (3)
10.6	5.0 (1.5)	7 (6)	5 (4)	30 (10)	18 (8)	24 (10)	19 (7)
12.8	5.9 (1.6)	11 (8)	6 (3)	37 (17)	20 (10)	25 (10)	21 (6)

Values in parentheses represent one standard deviation.

### Regression analyses

**Figure 3:**
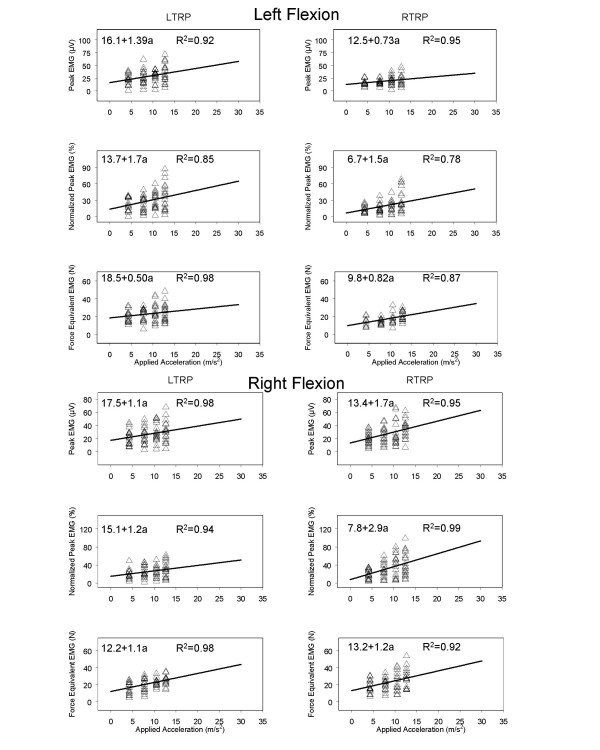
Trunk flexed to left and right. Extrapolated regression plots of the effect that applied acceleration has on the left and right trapezius muscles for the variables of peak electromyogram (EMG) (μV), normalized EMG (percentage of isometric maximal voluntary contraction), and force equivalent of EMG (N).

**Figure 4:**
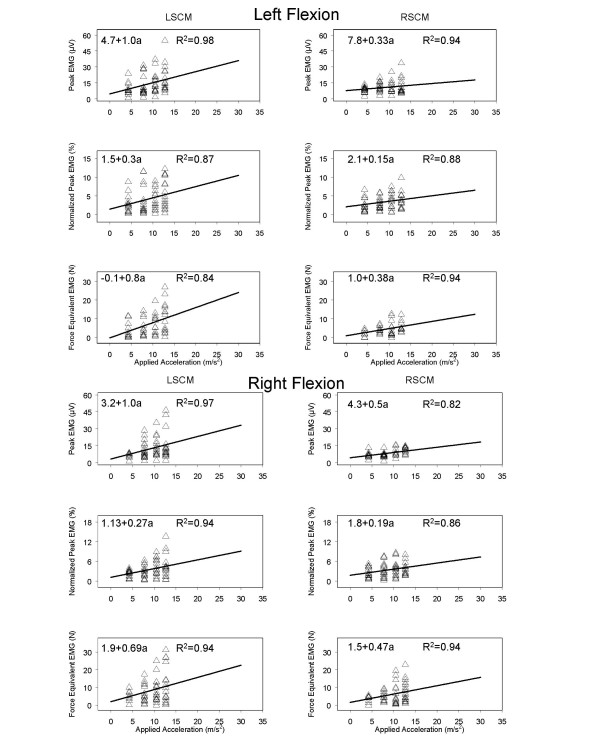
Trunk flexed to left and right. Extrapolated regression plots of the effect that applied acceleration has on the left and right sternocleidomastoid muscles for the variables of peak electromyogram (EMG) (μV), normalized EMG (percentage of isometric maximal voluntary contraction), and the force equivalent of EMG (N).

The applied acceleration, and the muscles examined had significant main effects on the peak EMG activity (p < 0.05) as shown in Table 2. We used a linear regression model to plot the available data and extrapolate from the experimental accelerations to accelerations on the order of 30 m/s^2^. Initially, regression analyses were performed only up to the maximal acceleration using a linear function. The kinematic variables of head displacement, velocity, and acceleration in response to the applied acceleration were calculated. Additionally, we also regressed the EMG magnitudes on acceleration. The responses of the left and right muscle groups were extrapolated to more than twice the applied acceleration value (see Fig. 3 and 4). It is of note that the EMG magnitudes remain low over this range compared to previous studies with the head and trunk in neutral posture [31].

**Table 2:** ANOVA table for Peak EMG (μV) by Muscles and Applied Acceleration.

		df	F	Sig.
Right Trunk Flexion	Accel	3	18.383	0.00
	Muscle	5	23.816	0.00
Left Trunk Flexion	Accel	3	12.296	0.00
	Muscle	5	53.261	0.00

At the time of impact, whiplash victims may be leaning forward or leaning over as a result of watching for traffic or speaking with other occupants, reaching for an object on the floor, et cetera. In the current study, having kept the impact direction constant, but varying trunk flexion to right or left we see that the muscles likely activated by holding this position (the ipsilateral trapezius), are most active and differ from their counterparts. Overall, however, the EMG activity is reduced if the subjects are "out-of-position" at the time of impact (the current study) compared to identical impact scenarios where the head and trunk are in neutral position. When the head was in neutral position in a previous study of right anterolateral impact [31], the left trapezius generated the greatest EMG, up to 83% of the maximal voluntary contraction EMG, and the left splenius capitis instead became more active and reached a level of 46% of this variable. As seen in this experiment, even the most active muscles do not exceed 44% of their maximal EMG contraction magnitude. The sternocleidomastoid muscles, by their attachment and action, are least likely to undergo eccentric contraction in the presence of what we expect is much less head-torso lag in the trunk -flexed posture. In contrast, the attachment and action of the trapezii, cervical extension being one action, are likely in a "pre-stretched" position in the trunk flexed posture with the subject looking downward. Even lower than expected head-torso lag in this posture is thus expected to generate more response and a higher likelihood of eccentric contraction in the trapezii than the sternocleidomastoids.

## Conclusion

It is suggested that the flexed trunk posture does not increase the likelihood of cervical muscle injury as compared to impacts with the trunk in neutral position, at least not for low-velocity impacts. Our findings are contrary to previous research findings [28]. Previous research, however, focused on dummy responses, which may explain the difference in our findings, and also some of the dummy experiments were of much higher velocity impacts. Nevertheless, symptoms are reported even after low-velocity impacts, and these lead to as many as 60% of injury claims [16]. With low-velocity impacts, one does not expect any significant rebounding of the subject back into the seat, and from our extrapolations, a trunk-flexed posture, assuming no bodily impact otherwise, does not otherwise appear to increase the risk of cervical muscle injury compared to occupant positioning in the neutral posture.

## Abbreviations

MVC (Maximal Voluntary Contraction); EMG (Electromyogram); cm (Centimetres); dB (decibels); C4 (fourth cervical vertebra); mV/g (Millivolts per gram); Hz (Hertz); kHz (kilohertz); g (acceleration due to gravity); m/s2 (metres per second per second); kg (kilograms); SCM (Sternocleidomstoid); TRP (Trapezius); SPL (Splenius capitis)

## Competing interests

The author(s) declare that they have no competing interests.

## Authors' contributions

SK made substantial contributions to conception and design, to acquisition of data, and analysis and interpretation of data, was involved in drafting the article and revising it critically for important intellectual content. RF made substantial contributions to analysis and interpretation of data, and was involved in drafting the article and revising it critically for important intellectual content. YN made substantial contributions to acquisition of data, and analysis and interpretation of data. EV made substantial contributions to analysis and interpretation of data. All authors read and approved the final manuscript.
